# Interventions for treating obesity in children

**DOI:** 10.1590/S1516-31802009000500015

**Published:** 2010-02-03

**Authors:** Hiltje Oude Luttikhuis, Louise Baur, Hanneke Jansen, Vanessa A. Shrewsbury, Claire O’Malley, Ronald P. Stolk, Carolyn D. Summerbell, Cochrane Heart Group

## Abstract

**BACKGROUND:**

Child and adolescent obesity is increasingly prevalent, and can be associated with significant short- and long-term health consequences.

**OBJECTIVE:**

To assess the efficacy of lifestyle, drug and surgical interventions for treating obesity in childhood.

**SEARCH STRATEGY:**

We searched CENTRAL on The Cochrane Library Issue 2 2008, MEDLINE (Medical Literature Analysis and Retrieval System), EMBASE (Excerpta Medica Databases), CINAHL (Cumulative Index to Nursing and Allied Health Literature), PsycINFO, ISI (Institute for Scientific Information) Web of Science, DARE (Database of Abstracts of Reviews of Effects) and NHS EED (National Health Service Economic Evaluation Database). Searches were undertaken from 1985 to May 2008. References were checked. No language restrictions were applied.

**SELECTION CRITERIA:**

We selected randomised controlled trials (RCTs) of lifestyle (i.e. dietary, physical activity and/or behavioural therapy), drug and surgical interventions for treating obesity in children (mean age under 18 years) with or without the support of family members, with a minimum of six months follow up (three months for actual drug therapy). Interventions that specifically dealt with the treatment of eating disorders or type 2 diabetes, or included participants with a secondary or syndromic cause of obesity were excluded.

**DATA COLLECTION AND ANALYSIS:**

Two reviewers independently assessed trial quality and extracted data following the Cochrane Handbook. Where necessary authors were contacted for additional information.

**MAIN RESULTS:**

We included 64 RCTs (5230 participants). Lifestyle interventions focused on physical activity and sedentary behaviour in 12 studies, diet in 6 studies, and 36 concentrated on behaviorally orientated treatment programs. Three types of drug interventions (metformin, orlistat and sibutramine) were found in 10 studies. No surgical intervention was eligible for inclusion. The studies included varied greatly in intervention design, outcome measurements and methodological quality.Meta-analyses indicated a reduction in overweight at 6 and 12 months follow up in: i) lifestyle interventions involving children; and ii) lifestyle interventions in adolescents with or without the addition of orlistat or sibutramine. A range of adverse effects was noted in drug RCTs.

**AUTHORS’ CONCLUSIONS:**

While there is limited quality data to recommend one treatment program to be favoured over another, this review shows that combined behavioural lifestyle interventions compared to standard care or self-help can produce a significant and clinically meaningful reduction in overweight in children and adolescents. In obese adolescents, consideration should be given to the use of either orlistat or sibutramine, as an adjunct to lifestyle interventions, although this approach needs to be carefully weighed up against the potential for adverse effects. Furthermore, high quality research that considers psychosocial determinants for behaviour change, strategies to improve clinician-family interaction, and cost-effective programs for primary and community care is required.

FURTHER INFORMATION:

Centro Cochrane do Brasil

Rua Pedro de Toledo, 598

Vila Clementino - São Paulo (SP) - Brasil

CEP 04039-001

Tel. (+55 11) 5579-0469/5575-2970

http://www.centrocohranedobrasil.org.br/

